# Drug-coated balloon therapy is more effective in treating late drug-eluting stent in-stent restenosis than the early occurring one—a systematic review and meta-analysis

**DOI:** 10.3389/fcvm.2023.1062130

**Published:** 2023-06-05

**Authors:** Péter Kulyassa, Marie Anne Engh, Péter Vámosi, Péter Fehérvári, Péter Hegyi, Béla Merkely, István Ferenc Édes

**Affiliations:** ^1^ Heart and Vascular Center, Department of Cardiology, Semmelweis University, Budapest, Hungary; ^2^ Centre for Translational Medicine, Semmelweis University, Budapest, Hungary; ^3^ Department of Biomathematics and Informatics, University of Veterinary Medicine, Budapest, Hungary

**Keywords:** in-stent restenois, drug coated balloon, drug eluting stent, recurrent revascularization, neointima proliferation, neoatherosclerosis

## Abstract

Drug-eluting stent in-stent restenosis (DES-ISR) remains one of the important assignments to be resolved in interventional cardiology, as it is present in 5%–10% of total percutaneous coronary intervention cases. Drug-coated balloon (DCB) utilization is promising, as it comes with long-term protection from recurrent restenosis in optimal conditions without the hazard of higher risk for stent thrombosis and in-stent restenosis. We aim to reduce the need for recurrent revascularization in DES-ISR, specifying the population in which the DCB therapy should be used. In this meta-analysis, the results of studies containing data on the time frame between drug-eluting stent implantation and the clinical presentation of in-stent restenosis and concomitant drug-coated balloon treatment were summarized. A systematic search was performed in Medline, Central, Web of Science, Scopus and Embase databases on November 11th, 2021. The QUIPS tool was used to assess the risk of bias in the included studies. The occurrence of a major cardiac adverse events (MACE) composite endpoint, containing target lesion revascularization (TLR), myocardial infarction, and cardiac death, and each of these separately, was assessed at 12 months after the balloon treatment. Random effects meta-analysis models were used for statistical analysis. Data of 882 patients from four studies were analyzed. Across the included studies, a 1.68 OR (CI 1.57–1.80, *p* < 0.01) for MACE and a 1.69 OR (CI 1.18–2.42 *p* < 0.01) for TLR were observed, both in favor of late DES-ISR. The main limitation of the study is the relatively low patient number. Nevertheless, this analysis shows the first statistically significant results for the effect of DCB treatment in the early or late presentation of DES-ISR. As to date, intravascular imaging (IVI) remains limitedly accessible, other landmarks as the time frame of in-stent restenosis development are to be pursued to advance therapeutic outcomes. In consideration of other biological, technical and mechanical factors, time frame of occurrence as a prognostic factor could reduce the burden of recurrent revascularization in patients at an already high risk.

**Systematic Review Registration**: identifier [CRD42021286262].

## 1. Introduction

Coronary heart disease affects approximately 126 million people worldwide (1). Alongside the preventive approach and medication-based treatment, in the event of hemodynamically significant stenosis of the epicardial vessels, percutaneous coronary intervention (PCI) is performed with drug-eluting stent (DES) implantation. The applied invasive proceedings are intended to reduce symptoms and/or improve prognosis. Although the eluted drug inhibiting cell proliferation is added on purpose to prevent in-stent restenosis from developing (ISR), DES-ISR is still observed in 5%–10% of total procedures based on implanted stent characteristics (2).

A variety of underlying pathophysiological processes can contribute to the development of ISR. Mechanical and technical factors include stent underexpansion prone to vessel calcification or multiple stent layers, stent fracture, stent undersizing, and geographic miss. Biological mechanisms of ISR mainly include neointimal hyperplasia and neoatherosclerosis (3–5). These may coexist; therefore, all possible factors should be identified and addressed properly. Neointimal hyperplasia is the intimal accumulation of smooth muscle cells and extracellular matrix. Neoatherosclerosis is characterized by an accumulation of lipid-laden foamy macrophages, with possible but not mandatory necrotic core formation, and neointima calcification (6, 7). Neoatherosclerosis with calcified parts can be challenging to manage, and this finding may rightly influence decisions regarding percutaneous intervention (8, 9).

Different types of DES-ISR (focal in-stent, focal peri-stent, and diffuse) may require a peculiar palette of treatment devices to attain optimal results. Some severely calcified lesions require thorough lesion preparation by cutting or scoring balloons, by rotational atherectomy or by intravascular lithotripsy. Two main treatment strategies emerged with favorable long-term outcomes in previous studies. Currently, the implantation of a new layer of DES appears to be a mildly superior option, yet it yields an elevated risk of acute stent closure, and stent thrombosis (10). Drug-coated balloon (DCB) therapy is a relatively novel modality, which, in addition to having about the same results as DES treatment, does not come with an elevated risk of such serious events. A recent publication suggests that tailored antiplatelet therapy is reducing the burden of recurrent revascularization after DES implantation. Besides the proven, there is a supposed additional clinical benefit of stronger agents when low bleeding risk is paired with low ischaemic risk (11).

The time course of DES-ISR after the index procedure appears to be dependent on the underlying stent type, and this may have relevance when patient follow-up is planned after coronary stent implantation. While bare metal stent (BMS) ISR was recognized to peak within the first six months after stent implantation, the incidence of DES-ISR appears to continue to increase steadily as a result of different mechanisms, including accelerated neoatherosclerosis, for several years (7, 12–14).

The changes of stent technologies led over the years to substantial changes in ISR pathomorphosis in comparison to the BMS era. The qualitative and quantitative representation of the three main components of ISR (neoatherosclerosis, VSMC, extracellular matrix) has changed since newer-generation DES ISR is often hypocellular and proteoglycan-rich, while in BMS-ISR VSMCs are predominantly present with a moderate proteoglycan content. Neoatherosclerosis is accelerated with first-generation DES, rare with BMS, and habitually develop over the long term with newer-generation DES, more often observed after one year. Late lumen loss with BMS often reaches a peak 6 to 8 months after implantation and then declines. Concerning DES, there is a slow and progressive neointimal buildup through 5 years following implantation.

One year from the index procedure is considered as a defining point to differentiate between early and late DES-ISR (15–17). It is based on OCT studies, where morphological findings differed between early and late (>1 year) second-generation DES ISR. Early ISR is more often associated with stent underexpansion and neointimal hyperplasia, while neoatherosclerosis prevails in late ISR (18). In a recently published study, neoatherosclerosis was the prevalent mechanism of ISR, with incidence ranging from 20% at 1 to 3 years and reaching above 70% at 7 years (19).

There are limited data showing that treatment with DCB might not be as effective in early DES-ISR (developing in <12 months) as in late DES-ISR (developing in >12 months) (20). As the current available literature takes the effect of time needed to develop in-stent restenosis only partially into consideration, there is a knowledge gap regarding this matter. The clinical relevance of measuring the time rather than performing IVI based revascularization is a question still waiting to be addressed. However, currently these additional proceedings of plaque visualization are more than doubling the costs of a PCI and are not accessible in most countries. The use of the target stent age could be an important measure determining the modality chosen in recurrent revascularization, and it could lead to a reduced burden of adverse events and possible accompanying complications. The timing of DES-ISR presentation could foreshadow a more aggressive nature of the vascular disease; nevertheless, it is not known whether early or late plaques would have better outcomes after DCB treatment. In late DES-ISR cases where neoatherosclerosis is commonly observed, progressively developing calcification is more prone to be present. It could lead to the attenuation of long-term lumen patency after recurrent revascularization (15, 17, 21).

Our hypothesis was that DCB is more effective in late DES-ISR than in the early one. The question to answer in this meta-analysis is whether or not the timing of DES-ISR (early vs. late) affects the outcomes of DCB treatment. We aim to reduce the need for recurrent revascularization in DES-ISR, specifying the population in which the DCB therapy should be used. We assessed the available literature and summarized quantified results.

## 2. Methods

### 2.1. Search strategy and selection criteria

This systematic review is being reported in accordance with the PRISMA (Preferred Reporting Items for Systematic Review and Meta-Analyses) 2020 guideline (22). A systematic search was performed in five databases on November 11th, 2021, and altogether 832 articles were found. The databases included Medline (181), Central (117), Web of Science (214), and Scopus (192), with the search key: (early OR late) AND (in-stent restenosis OR ISR) AND (drug coated balloon OR DCB OR paclitaxel coated balloon OR PCB OR sirolimus coated balloon OR SCB) and Embase (128) with the search key: (early OR late) AND (“in-stent restenosis” OR ISR) AND (“drug coated balloon” OR DCB OR “paclitaxel coated balloon” OR PCB OR “sirolimus coated balloon” OR SCB). Studies examining cases of DES-ISR and DCB as treatment modality were eligible for the analysis. The time frame between DES implantation and DCB treatment was needed to determine DES-ISR presentation timing. In the absence of the latter, requests were sent to the authors for patient level data. Data pools containing the treatment of lesions with modalities other than DCB were not excluded when separate group analysis and detailed patient characteristics were disclosed. Two independent reviewers assessed the available articles. The citations were managed in Endnote ×9 by Clarivate Analytics. After automatic and consecutive manual duplicate removal, the selection was done in a two-stage process (Title-abstract, Full-text). Cohen's kappa coefficient (*κ*) was calculated after each selection step for inter-reviewer reliability measurement (23). Disagreements were resolved by a third author. The data were collected from the full-text articles and conference abstracts by two independent reviewers systematically with the use of pre-planned data extraction tables. These were constructed under the surveillance of our statistical team and with the consent of all authors of this article.

### 2.2. Outcomes and extraction

Primary and secondary outcomes were defined. All outcomes were searched for in all published articles conformant with our selection criteria. Insufficient or compromised data were not included in the analysis. The primary outcome was the major adverse cardiac event (MACE), which includes three subcomponents: target lesion revascularization (TLR), cardiac death (CD), and myocardial infarction (MI). These outcomes were also examined separately. The secondary endpoints were target lesion thrombosis, target vessel revascularization, and late lumen loss, which were not found in any of the articles. General patient characteristics like age, sex, smoking status, and medical comorbidities like hypertension, diabetes mellitus, dyslipidemia, chronic kidney disease, and earlier cardiovascular events were registered if available. There were no assumptions made for missing values from accessible data. Subgroup analysis was planned to be performed if at least three studies are present in a given subgroup.

### 2.3. Risk of bias assessment

Two independent authors assessed the risk of bias of the included trials separately. The QUIPS tool was used as an assessment guide for prognostic studies. It investigates six domains: study participation, study attrition, prognostic factor measurement, outcome measurement, study confounding, and statistical analysis and reporting (24). The disagreements in assessment grades between the studies were resolved by consensus of the two authors. There was no automation used in the process.

### 2.4. Statistical analyses

The odds ratio with 95% CI was used for the effect measure; to calculate the odds ratio, the total number of patients in each group, and those with the event of interest were extracted from each study. Raw data from the selected studies were pooled using a random effects model with the Mantel-Haenszel method and the Hartung-Knapp adjustment (25, 26). To estimate *τ*2 the Paule-Mandel method was utilized together with the Q profile method to calculate the confidence interval of *τ*2 (27, 28). For publication bias evaluation, a funnel plot of the logarithm of effect size and comparison with the standard error for each trial was used. Statistical heterogeneity across trials was assessed by means of Cochrane Q test, and the I2 values (29). Outlier and influence analyses were carried out in light of the recommendations of Harrer et al. (28) and Viechtbauer and Cheung (30). Publication bias was visually assessed with a funnel plot, as the number of studies was low for all outcomes.

## 3. Results

### 3.1. Selection of articles

**Figure 1:**
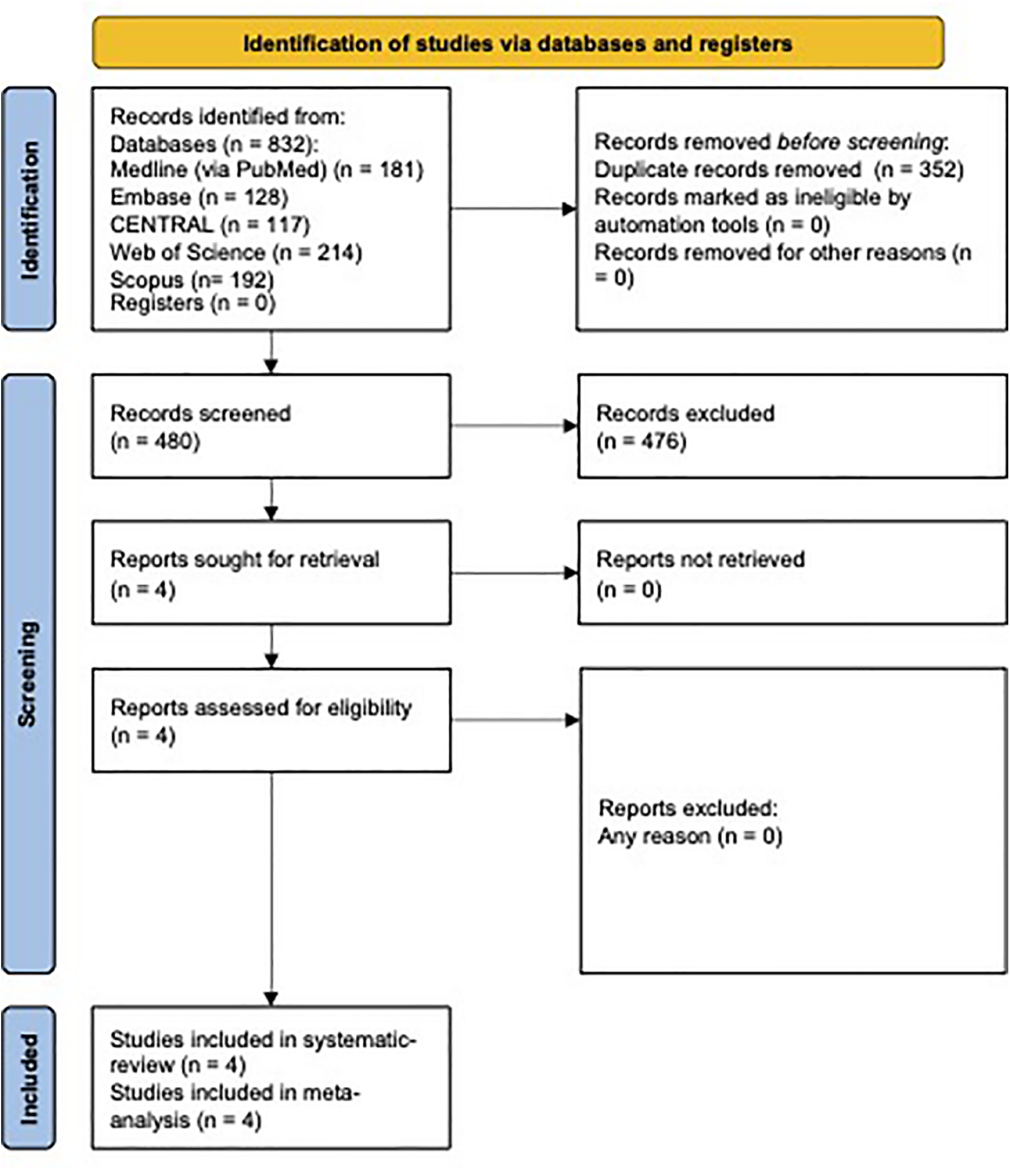
Flow diagram of systematic search and article selection.

There were 832 studies in the initial search, four of which were selected for full-text review. All four articles were included in further inquiries. The PRISMA flow diagram of the systematic search and the selection process can be seen in Figure 1 (31).

### 3.2. Major cardiovascular adverse events

**Figure 2:**
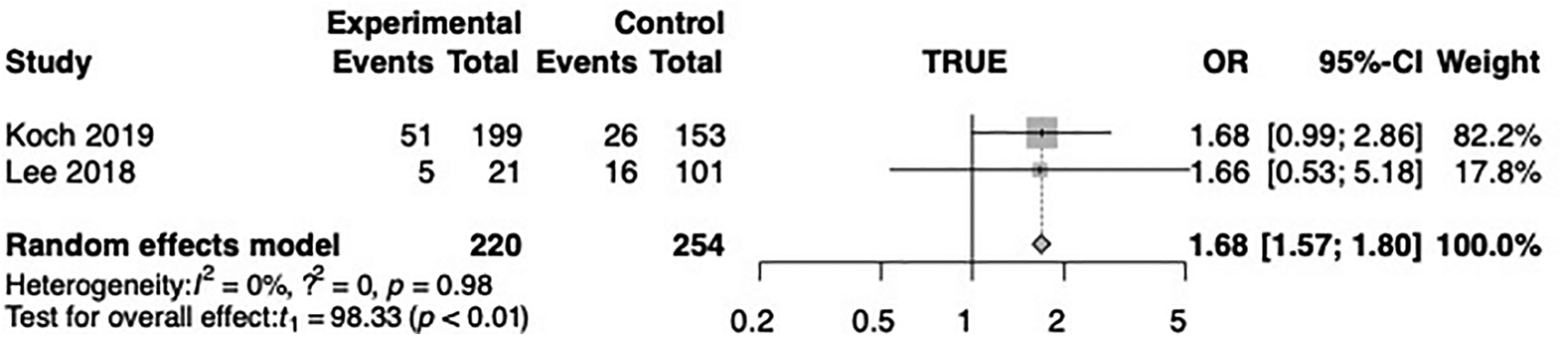
Forest plot of major cardiac adverse events (MACE).

In the meta-analysis, the MACE OR was 1.68 (CI 1.57–1.80, *p* < 0.01) in the early vs. late DES-ISR group. This outcome was identified in two studies (20, 32). In connection with MACE, one study (20) compared early DES-ISR patients with late DES-ISR patients and found that the former group showed a significantly higher risk (25.9% vs. 17.0%; *p* = 0.04) 12 months after DCB treatment, OR was 1.68 (CI 0.99–2.86). According to one study (32) the incidence of MACE in the early DES-ISR group 12 months after DCB treatment was 5 and in the late DES-ISR group 16 (23.8% vs. 15.8%; *P* < 0.01), the OR was 1.66 (CI 0.53–5.18) 12 months after DCB treatment. The summary of the results can be seen in Figure 2.

### 3.3. Target lesion revascularization

**Figure 3:**
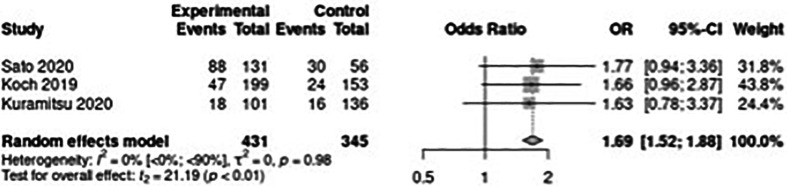
Forest plot of target lesion revascularization (TLR).

When TLR was assessed across the included studies, a 1.69 OR (CI 1.18–2.42, *p* < 0.01) was observed. TLR was found in three studies. In one (20), TLR was 47 (24.0) in the early DES-ISR group and 24 (15.7) in the late DES-ISR group 12 months after DCB treatment, giving an OR of 1.66 (CI 0.96–2.78). In 187 ISR cases in the study by Sato et al. (33), the overall TLR rate was 0.33 (128), which distributed as 0.40 (88) in the early and 0.16 (30) in the late DES-ISR group at a median of 12 months after DCB treatment, the OR was 1.77 (CI 0.94–3.36). Kuramitsu et al. (34) found that the TLR rate was significantly higher in the early ISR group than in the late ISR group (30.0% vs. 18.3%, *p* = 0.035) 12 months after DCB treatment, which resulted in an OR of 1.63 (CI 0.78–3.37). The one-year outcomes from the Kuramitsu study were extracted from the figure “cumulative incidence of TLR after DCB” in the abstract with the use of Web Plot Digitizer (35). The TLR endpoint in one study (32) was not fit for data extraction using this method, as the figure and the numbers showed ambiguity. Based on this, the author group decided not to include this study in the statistical analysis. The summary of the results can be seen in Figure 3.

### 3.4. Cardiac death

One paper reported (20) three (1.5%) cardiac deaths in the early DES-ISR group and none in the late (*p* = 0.82). In another (32) there were no cardiac deaths in either of the groups. In the studies by Sato et al. and Kuramitsu et al. there was no information disclosed on the matter.

### 3.5. Myocardial infarction

In one study there were six (3.0%) myocardial infarctions in the early and two (1.3%) in the late DES-ISR group (*p* = 0.29). In one other (32), data were not extractable for one year. In the studies by Sato et al. and Kuramitsu et al. there were no data published for this outcome.

### 3.6. Target lesion thrombosis

In Koch et al. (20), the rate of target lesion thrombosis was low. There was one event in the early DES-ISR group (0.5%) and no events in the late DES-ISR group (0.0%, *p* = 0.90). In the studies by Lee et al. (32) and Sato et al. (33) there were no data published about the outcome. Kuramitsu et al. (34) had low event numbers (3.3% in early DES-ISR vs. 0.8% in late DES-ISR group, *p* = 0.20) in two years; however, the one-year data were not extractable from the text, and no graphs were available on this outcome either.

### 3.7. Target vessel revascularization

There were no data disclosed in the included studies about this outcome. No further data were provided on this subject by the contacted authors.

### 3.8. Late lumen loss

There were no data disclosed in the included studies about this outcome. No further data were provided by the contacted authors on this subject.

### 3.9. Subgroup analysis

There were not enough number of studies as planned in the protocol for subgroup analysis, therefore it was not performed.

### 3.10. Summary of included articles

Sato et al. included 187 patients who encountered ISR after the implantation of second-generation DES. Their patients received target lesion revascularization (TLR) with DCB angioplasty. They received coronary angiography after a median of one-year of follow-up or symptomatic reasons. Some of the patients encountered recurrent ISR and received further TLR. As the primary endpoint, the recurrent TLR was assessed. The cutoff between early and late ISR was determined by Receiver Operating Characteristic curve (*n* = 131). The early ISR group had ISR within 1.6 years, the late ISR group (*n* = 56) had the occurrence at more than 1.6 years after the index procedure.

Lee et al. included 122 patients (122 ISR lesions), treated with DCB under optical coherence tomography (OCT) examination before and after DCB, and categorized ISR (<12 months; E-ISR; *n* = 21) and late ISR (≥12 months; L-ISR; *n* = 101). Associations between OCT-based neointima characteristics and the period of ISR and also clinical outcomes after DCB were evaluated. Major adverse cardiac events (MACE) were a composite of cardiac death, non-fatal myocardial infarction, or target lesion revascularization (TLR). Although the data for MACE could be seen in the graphical interpretation for one year of follow-up, the presented numbers and the figure could not be definitively determined; therefore, it was not included in that outcome.

Koch et al. published a pooled analysis including patients with DES-ISR assigned to treatment with DCB in the setting of the ISAR DESIRE 3 and 4 trials. According to the time of ISR occurrence after DES implantation clinical outcomes were evaluated, in patients presenting with early (≤12 months) vs. late DES-ISR (>12 months) undergoing treatment with DCB. The primary endpoint of this analysis was major adverse cardiac event (MACE), defined as the combined incidence of death, myocardial infarction and target lesion revascularization (TLR) 12 months after DCB treatment. Secondary endpoints included the incidence of death, myocardial infarction, TLR and target lesion thrombosis 12 months after DCB treatment. The analysis included 352 patients, 199 patients presented with early-ISR, 153 patients with late-ISR.

Kuramitsu et al. disclosed the results of a total of 239 consecutive patients with 291 ISR lesions after newer-generation DES were treated with DCB. According to the timing of ISR, patients were divided into the two groups: early ISR group (<1 year after the index procedure, *n* = 103) and late ISR group (≥1 year after the index procedure, *n* = 136). The cumulative incidence of TLR and stent thrombosis (ST) within the first two years after DES implantation were assessed. No significant differences in baseline patient and lesion characteristics were found in the early and late ISR groups except for a higher prevalence of hemodialysis in the early ISR group (33.0% vs. 15.4%, *p* = 0.002). Patient, intervention and outcome characteristics are summarized in Table 1.

**Table 1:** Patient, intervention and outcome characteristics of included studies.

Study	Early/late definition	Exposed group (early DES-ISR) population	Comparison group (late DES-ISR) population	Outcome assessment time	MACE intervention arm	MACE control arm	TLR intervention arm	TLR control arm	Inclusion criteria
Sato	Average 19 months	131	56	Median 1 year or based on symptom occurrence	Not published	Not published	88	30	DCB treatment of the lesion
Koch	12 months	199	153	1 year based on symptom occurrence	51	26	47	24	DCB treatment of the lesion
Lee	12 months	21	101	5 years based on symptom occurrence	5	16	Not published	Not published	DCB treatment of the lesion
Kuramitsu	12 months	101	136	2 years based on symptom occurrence	Not published	Not published	18	16	DCB treatment of the lesion

### 3.11. Risk of bias and heterogeneity assessment

**Figure 4:**
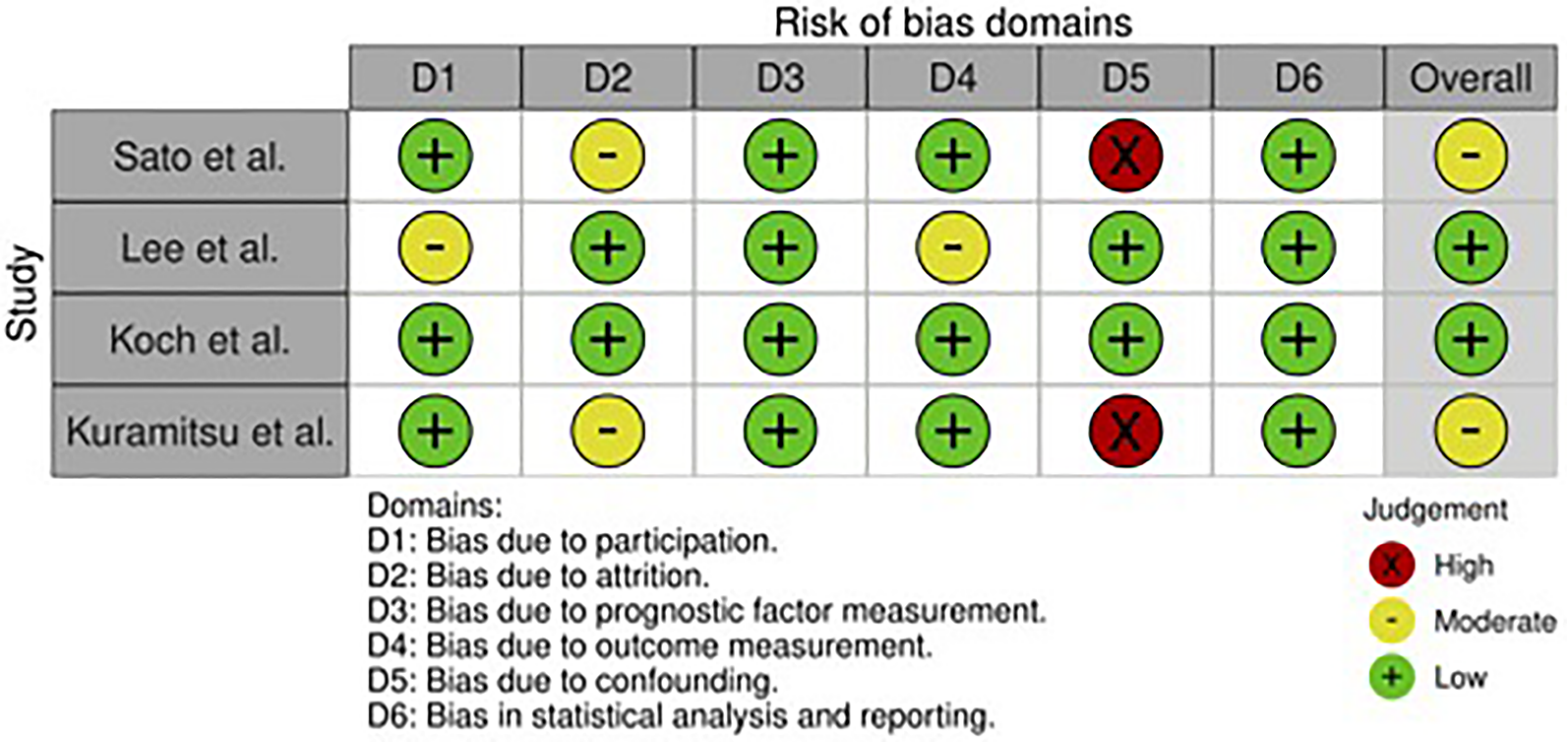
Risk of bias assessment across included studies.

The overall risk of bias was low in two studies and moderate in the other two. The assessment is summarized in Figure 4. The included full-text articles discussed patient characteristics. As patient and publication numbers were limited, no further heterogeneity assessment was performed.

## 4. Discussion

### 4.1. Main findings

This meta-analysis is the first of its kind to assess the effect of the early or late presentation of DES-ISR, done here on the basis of four trials. In the early DES-ISR population treated with DCB, the risk of having repeated revascularization on the given artery segment is 69% higher than in late DES-ISR. Patients presenting with early DES-ISR had 68% higher chance of suffering myocardial infarction, cardiac death or having repeated revascularization compared to patients presenting with late DES-ISR for DCB treatment.

For the TLR outcome, the events were assessed differently in the trials. In the study by Sato et al., at median one year, a repeated coronary angiography was performed. The authors did not disclose any further details on the timing of the coronary patency reassessment. In the study by Koch et al. a one-year follow-up was allowed, and if symptoms occurred, repeated coronary angiography and revascularization were performed based on the current guidelines of ISR revascularization. Lee et al. and Kuramitsu et al. followed the same process based on the available data, but with extended follow-up time. However, the precise event count at one year follow-up could be extracted only from the article by Kuramitsu et al. but not from that by Lee et al. as discussed earlier. Further patient characteristics assessment was not possible based on the low number of included trials. This enhances the possibility of confounding bias present in the outcomes. The reporting bias was medium to low in the included studies. The certainty of proper outcome measurement is high as the nature of the observed outcomes is principally clinically driven. There are no earlier data on this subject, as DES-ISR is observed to have a relatively low frequency after initial revascularization.

It is worth mentioning that Sato et al. found on the basis of a multi-variable analysis that late ISR (Hazard ratio: HR 0.44, 95% CI 0.29–0.67, *p* < 0.001), and restenosis type of Mehran Ic (focal restenosis) (HR 0.57, 95% CI 0.34–0.97, *p* = 0.04) were the independent factors related to the recurrent TLR. According to Kuramitsu et al., hemodialysis (hazard ratio [HR] 3.03, 95% confidence intervals [CI]: 1.68–5.38, *p* < 0.001) and diffuse ISR pattern (HR 2.39, 95% CI: 1.34–4.19, *p* = 0.004) were predictors of TLR. In a multivariate analysis by Koch et al. including diabetic status, clinical presentation, previous coronary bypass graft, and diameter stenosis after DCB-treatment, the adjusted hazard ratio showed significantly higher risk for MACE of early DES-ISR as compared to late DES-ISR (HRadj = 1.8, [95% CI = 1.1–3.0], *p* = .02). These observations are parallel with the theory that the simpler and the later presenting subtypes of ISR by patients with less cardiovascular risk are prone to appear with better outcomes after recurrent revascularization whereas stent thrombosis rate did not significantly differ between groups (3.3% vs. 0.8%, *p* = 0.20) (36).

### 4.2. In-stent restenosis characteristics

ISR develops in two distinct, but often mixed histopathological forms. Neointimal hyperplasia is characterized by the migration and proliferation of vascular smooth muscle cells (37). The forming of a novel fibroatheroma within the stent struts, also called neoatherosclerosis, is a longer-term process, promoting late DES-ISR and very late stent thrombosis (38). It is characterised by an accumulation of lipid-laden foamy macrophages, potential calcification within the neointima with or without necrotic core formation (6). Neoatherosclerotic ISR appears to develop in tandem with native atherosclerotic disease progression. Is suggests similar underlying pathomechanisms, but in comparison, neoatherosclerosis demonstrates an accelerated course to *de novo* atherosclerotic CAD (39).

The ISR IVI appearance is most likely representative of the underlying histopathology. A homogenous tissue appearance on OCT has been shown to correspond to neointima and fibrous connective tissue as a result of smooth muscle cell proliferation. Conversely, heterogenous tissue patterns are associated with increased display of fibrin depositions and loose connective tissue (40). In DES ISR, different types of heterogenous patterns are observed, which seem to gradually change in time. In an observational OCT study thin cap fibroatheroma-like pattern image and intra-intima microvessels were increasing from the early to the late phase, the speckled pattern image rarefacted from the early to the late phase (16).

BMS-ISR and different generations of DES-ISR shows different histopathological and IVI characteristics. BMS-ISR is characterized by homogeneous tissue rich in smooth muscle cells, whereas DES restenosis is more often hypocellular and proteoglycan-rich. A key determining factor in the difference in OCT findings observed between BMS-ISR and DES-ISR appears to be the timing of the ISR progression. As studies pointed out, neoatherosclerosis is more likely to be present in the setting of DES implantation, where the released anti-proliferative agents delay vascular healing, promoting the formation of atheromas (36). In a human pathological study which examined first generation DES, neoatherosclerosis occured more frequent and earlier in DES compared to BMS (38).

Studies also suggest a different time course and different morphological characteristics in first and second generation DES. A human autopsy study found that second generation DES demonstrated greater strut coverage with less inflammation, less fibrin deposition compared to first generation DES. Nevertheless, the observed frequencies of neoatherosclerosis in the two groups were comparable (41). An OCT-driven observational study found that in second generation DES a heterogeneous pattern was prevalent both before and after 1 year. Neoatherosclerosis was more common in the early period in first generation DES, but after one year, was more prevalent in second generation DES (17). It has been also demonstrated that OCT findings suggestive of neoatherosclerosis seem less common in early DES-ISR than in late DES-ISR (15, 17, 42).

### 4.3. Clinical implications

According to Lee et al. the incidence of MACE was significantly higher for lesions with a heterogeneous than with a non-heterogeneous neointima (43.7% vs. 19.6%; *P* = 0.018), but it was not significantly associated with neoatherosclerosis (33.4% vs. 18.4%; *P* = 0.168). This parallels the findings of this meta-analysis that early presenting DES-ISR tends to display heterogenous tissue characteristics on IVI and is more resistant to recurrent revascularization with DCB (32). Expert consensus and guidelines recommend (Class IIa, Level B) the use of IVI to assess ISR (43, 44). However, it is still not accessible for all-case utilization based mainly on financial reasons. If IVI is not available in the decision making, after consideration of the earlier described mechanisms of DES-ISR, all gathered information should be used to tailor the chosen therapeutic approach.

The presence of a metal scaffold brings some additional considerations compared with *de novo* disease, and persistent issues leading to the original stent failure may need to be identified and addressed to avoid recurrence. It is important to note that in the setting of ISR, the following need of repeated revascularization will be impacted by not only the choice of treatment modality but also by extrinsic mechanical factors. A significant proportion of ISR lesions is associated with stent underexpansion, which may itself be secondary to vessel calcification. In addition, calcified neoatherosclerotic ISR can also result in specific challenges with respect to achieving a maximal acute gain.

### 4.4. Strengths and limitations

The results show the first statistically significant evidence that the early presentation of DES-ISR may predict worse outcomes after DCB revascularization. The results show a marked difference between the two groups despite the relatively low study and patient number. A significant proportion of aligned patient data is missing. This is a considerable limitation for the implementation of the results of this analysis, also shedding light on the need for further research on this subject. With the currently used generation of DES, there is limited evidence of long-term results. It is important to consider that the incidence of ISR may also be dependent on the nature of the follow-up, with increased identification of “silent” ISR in patients who have undergone a stent implantation and are reassessed without the presence of recurring symptoms. The different time frames used to determine early and late DES-ISR are considered a minor limitation of the meta-analysis, given the tendency for worse outcomes by developing DES-ISR earlier. There are no data on the different types of the second-generation DES involved in the study.

### 4.5. Conclusion

The age of the implanted stent could be an important prognostic factor in the case of DES-ISR. If other biological, technical, and mechanical factors are taken into consideration, it may assist the optimal choice of a treatment device in this patient population at high risk for more repeated revascularization. In order to verify this hypothesis, more clinical trials in randomized fashion are needed to be carried out on this subject. If IVI is available and the clinical scenario allows its use, then that is the preferable method for guiding therapy of ISR lesions. However, it remains limited in accessibility, and other relevant information regarding lesion characteristics seems worth pursuing. Therefore, further data collection on DES-ISR presentation timing and its effects on outcomes is also required.

## Data Availability

The original contributions presented in the study are included in the article, further inquiries can be directed to the corresponding author.
